# GC-MS-based fecal metabolomics reveals gender-attributed fecal signatures in ankylosing spondylitis

**DOI:** 10.1038/s41598-019-40351-w

**Published:** 2019-03-07

**Authors:** Zhixing He, Mingzhu Wang, Haichang Li, Chengping Wen

**Affiliations:** Institute of Basic Research in Clinical Medicine, College of Basic Medical Science, Zhejiang Chinese Medical University, Hangzhou, 310053 China

## Abstract

Ankylosing spondylitis (AS) has different clinical features in males and females. Fecal metabolites play significant roles in AS disorders. This study aimed to reveal gender-attributed fecal signatures of AS. Fecal samples from 87 cross-sectional individuals (healthy controls: 20 males, 18 females; AS patients: 26 males, 23 females) were analyzed by gas chromatography-mass spectroscopy (GC-MS). Partial least squares discriminant analysis (PLS-DA) was used to reveal differences in the fecal signatures of AS between males and females. Fecal signatures were defined by the significantly different fecal metabolites between AS patients and healthy individuals. Therefore, different fecal signatures of male and female AS patients were defined as gender-attributed fecal signatures. Male-specific fecal signatures in AS patients were steroid compounds, including cholestan-3-ol, tocopherol, stigmastan-3,5-diene, cholest-3-ene, cholest-4-en-6-one and 1-heptatriacotanol. Female-specific fecal signatures were ergost-5-en-3-ol, acetate and D-myo-Inositol. Gender-attributed fecal signatures of AS further reveal differences between males and females in terms of AS features.

## Introduction

Ankylosing spondylitis (AS) is a chronic rheumatic disorder characterized by inflammation of the spine and sacroiliac joints. The disease can cause altered physical outcomes such as reduced spinal mobility, fatigue, sleep disturbances and psychological consequences such as depression, anxiety and stress^1^. In addition, multiple coexisting comorbidities are frequent in AS, especially in patients with cardiovascular disease^2^. Currently, the occurrence of AS is considered to be triggered by genetic, infectious and endocrine factors^3^.

Over the years, AS has been considered to be a disease occurring predominantly in males, with an estimated male to female ratio of 2-3:1^4^. Additionally, there are some differences in the clinical features of AS between males and females. Compared with male patients, female patients were found to have a longer delay in diagnosis and a greater age at disease onset^4,5^. It has also been observed that female AS patients present a more active disease^6^, a higher burden of disease^5^, and more frequent peripheral involvement than do male AS patients^7,8^. With respect to clinical therapy, female patients are positively associated with the use of conventional disease-modifying antirheumatic drugs (DMARD) therapy, while male patients are associated with the use of biologic therapy^9^. Therefore, further research is necessary to better reveal differences between male and female AS patients.

Increasing evidence has indicated that genetic risk factors and pathogenesis are similar in AS and inflammatory bowel disease (IBD)^10^. Frequently, AS patients suffer from severe intestinal inflammation characterized by cytokine overexpression^11^. Therefore, AS is closely related to intestinal disorders involving gut microbiota and fecal metabolites. Alterations in gut microbiota have been associated with the development of AS^12,13^ and HLA-B27^14^. The ^1^H NMR spectroscopy of fecal metabolites showed that AS patients have significantly decreased levels of butyrate, propionate, methionine and UDP-glucose and increased levels of fumarate, glucose, taurine, tryptophan and threonine^15^. The cross talk between gut microbiota and intestinal cells is mediated by intestinal metabolites. Therefore, characterization of intestinal metabolites may be important for revealing different AS conditions in males and females.

In this study, GC-MS was used to investigate fecal metabolite profile of AS patients and healthy individuals in a cross-sectional study. The overall goal of this study was to ascertain whether the fecal signatures of AS were associated with gender.

## Materials and Methods

### Patient population

After informed consent was provided, a total of 87 individuals (49 AS patients and 38 healthy individuals) were enrolled in this study. AS was diagnosed on the basis of the modified New York criteria for AS^16^. There was a consistent male-to-female ratio between AS patients (26 male, 23 female) and healthy controls (20 male, 18 female) (Table 1). In addition, the age and body mass index (BMI) were also matched between AS patients and healthy individuals (HDs) (Table 1). In addition, HLA-B27 had a strong positive correlation with AS in all participants (Table 1). Erythrocyte sedimentation rate (ESR) and C-reactive protein (CRP) values were significantly higher in AS patients than in HDs (Table 1). In addition, the bath Ankylosing Spondylitis Disease Activity Index (BASDAI) scores of AS patients were calculated. Regardless of AS or healthy status, there were no significant differences in the above clinical information between males and females.

**Table 1:** Demographic and clinical chemistry characteristics of human subjects.

Characteristics	Heathy control			Ankylosing spondylitis		
	Total	Male	Female	Total	Male	Female
Gender	38	20	18	49	26	23
Age mean ± SD	43.1 ± 8.5	43.29 ± 9.0	43.0 ± 8.2	43.0 ± 9.6	42.2 ± 10.5	44.1 ± 8.4
[min, max]	[24, 56]	[24, 56]	[24, 55]	[24, 58]	[24, 58]	[24, 57]
BMI mean ± SD	20.8 ± 5.2	21.6 ± 6.3	19.8 ± 5.0	21.3 ± 4.7	21.8 ± 7.2	20.3 ± 5.6
[min, max]	[17.2, 25.5]	[18.1, 25.5]	[17.2, 24.9]	[16.6, 27.5]	[19.5, 27.5]	[16.6, 25.6]
HLA-B27 (+/−)	6/32	3/17	3/15	45/4	23/3	22/1
CRP mean ± SD	4.2 ± 1.9	5.1 ± 1.4	3.8 ± 1.7	8.7 ± 5.2	8.9 ± 4.8	8.3 ± 5.6
[min, max]	[0.5, 6]	[0.5, 6]	[0.7, 5.6]	[2, 37]	[5, 37]	[2, 35]
ESR mean ± SD	8.5 ± 4.2	8.4 ± 3.7	9.0 ± 3.8	13.6 ± 8.7	12.3 ± 8.3	14.5 ± 7.9
[min, max]	[1, 15]	[3, 15]	[1, 12]	[5, 42]	[5, 42]	[6, 32]
BASDAI ± SD	—	—	—	3.7 ± 2.1	3.6 ± 2.2	3.9 ± 2.1
[min, max]				[0.9, 8.8]	[0.9, 8.6]	[1.2, 8.8]

All participants with gastrointestinal tract disorders and those undergoing treatment with antibiotics, probiotics or drugs within one month prior to the stool collection were excluded. Patients with severe systemic diseases or hepatitis were excluded. A dietary questionnaire that recorded the complete diet information and dietary habits was completed. This questionnaire was used to exclude individuals who had specific dietary habits such as alcohol consumption or a completely vegetable-based diet. In this study, all experimental protocols involving humans were approved by the Ethics Committee of Zhejiang Chinese Medical University.

### Sample collection and preparation

Each participant was given a sample collection kit with instructions. One fecal sample was collected from each subject; placed in a sealed insulated container; immediately placed in ice; and subsequently delivered to the laboratory within 2 h, where it was stored at −80 °C.

Fifty milligrams of feces was used for the extraction procedure and extracted with 800 μL methanol. Then, 10 μL internal standard (2.9 mg/mL, DL-o-chlorophenylalanine) was added. All samples were ground to a fine powder using a grinding m at 65 HZ for 90 s. After grinding, the samples were vortexed for 30 s and centrifuged at 12000 rpm at 4 °C for 15 min. Then, 200 μL supernatant was transferred to a vial for concentration by centrifugation. The samples were derivatized by adding 30 μL methoxyamine hydrochloride (20 mg/mL) in pyridine and shaking them for 90 min at 37 °C, after which they were incubated for 16 h at room temperature. The samples were then trimethylsilylated by adding 30 μL BSTFA and incubating for 1 h at 70 °C; samples were then prepared for GC-MS analysis.

### GC-MS analysis conditions

Metabolic profiling of fecal samples was acquired by an Agilent 7890 A/5975C GC-MS (Agilent Technologies, Santa Clara, CA, USA). Separation was performed by using a 30 m × 0.25 mm × 0.25 μm HP-5MS fused silica capillary column (Agilent J&W Scientific). The sample injection volume was 1 μL with a split ratio of 10:1. The injector, ion source and quadrupole rod temperatures were 280 °C, 230 °C and 150 °C respectively. The flow rate of the carrier gas, high-purity helium (>99.999%), was 1.2 mL/min. The GC oven temperature program consisted of 80 °C for 2 min, after which the temperature ramped to 330 °C at 10 °C/min, and held steady for 6 min. Mass spectra were acquired at ascan speed of 2 spectra per second after a solvent delay of 4.8 min, and the mass scan range was set at m/z 50–550. Fecal samples were analyzed randomly.

### Data processing and statistical analysis

Raw GC-MS mass spectra were converted to CDF format files and subsequently processed using XCMS in R software as previously described^17^. XCMS could be employed for preprocessing automatically, including raw signal extraction, data baseline filtering, peak identification, and integration. After alignment with the statistical comparison component, the “.CSV” file was obtained with four dimension data sets including sample information, retention time, the mass-to-charge ratio and peak intensity. Identification of metabolites was conducted using the Automatic Mass Spectral Deconvolution and Identification System (AMIDS), which was searched against commercially available databases such as the National Institute of Standards and Technology (NIST) and Wiley libraries. Metabolites were identified by comparison of mass spectra and retention indices to the spectral library using a match value greater than 700. The signal integration area of each metabolite was normalized to the internal standard (DL-o-chlorophenylalanine) for each sample.

For multivariate statistical analysis, the XCMS output was further processed using Microsoft Excel (Microsoft, USA). The normalized data were transformed using SIMCA-P 11.0 software (Umetrics AB, Umea, Sweden) for principal component analysis (PCA) and partial least Squares-discriminant analysis (PLS-DA). PCA and PLS-DA were applied to the data after mean-centering and unit variance scaling (UV scaling). These analyses employed a default seven-fold internal cross validation from which the R^2^X and Q2 (goodness of prediction) values, representing the total explained variance and the model predictability, respectively, were extracted.

The variable importance in projection (VIP) values of all the metabolites from the PLS-DA model was taken as criteria to find the variable importance of differential metabolites. Those variables with a VIP >1.0 and a *p*-value < 0.05 were considered relevant for group discrimination. The statistical significance between two groups was evaluated by a univariate Student’s t-test. Following statistical analyses with multiple comparisons, p value were adjusted using the Benjamini-Hochberg method to control the false discovery rate (FDR). An adjusted *p* value of 0.05 was used as a statistically significant cutoff.

### Compliance with Human Studies and Ethical standards

All procedures performed in studies involving human participants were in accordance with the ethical standards of the institutional, national research committee and with the 1964 Helsinki declaration and its later amendments. Informed consent was obtained from all individual participants included in the study. All procedures involved humans in this study were approved by the Ethics Committee of Zhejiang Chinese Medical University.

## Results

### Metabolic profiles in fecal extracts

Typical GC-MS spectra of fecal extracts obtained from AS patients and HDs are shown in Fig. S1. Two thousand three hundred forty-two features were obtained from GC-MS spectra. The endogenous metabolites were then identified by similarity matching with the NIST05 mass spectra. Identified metabolites with a match value greater than 700 were used for subsequent analysis. As shown in Table S1, 84 metabolites were confirmed on the basis of their retention and MS fragmentation behavior, mainly including alkanes (11 identified), alkenes (2 identified), sugars (13 identified), organic acids (35 identified), and cholesterols (7 identified). To obtain detailed differences between groups, multivariate data analysis of these GC-MS profiles was performed.

### PCA analysis

**Figure 1:**
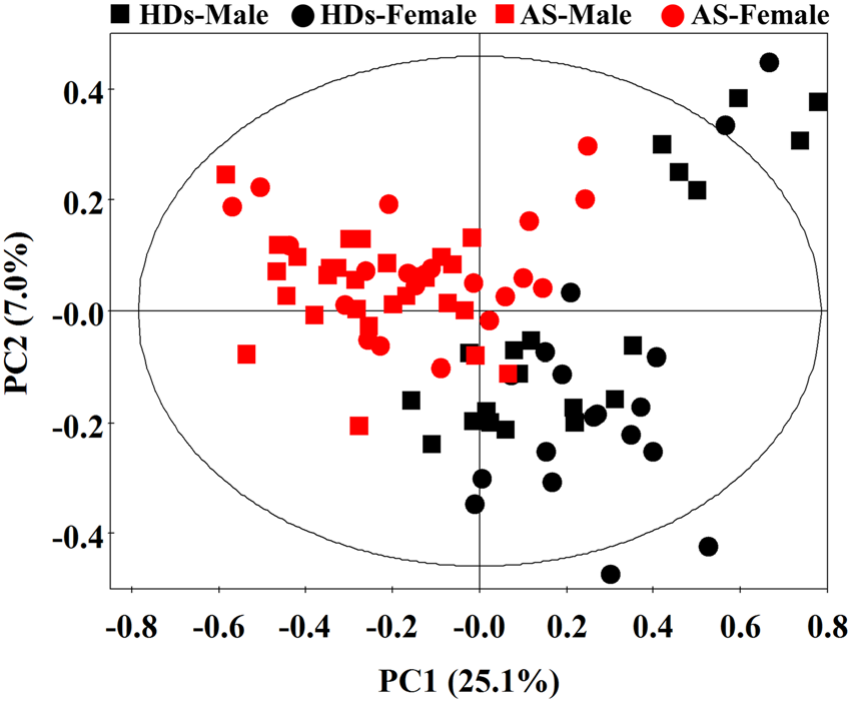
PCA score plots derived from GC-MS data for fecal extracts obtained from healthy individuals (male: *black box*; female: *black dots*) and AS patients (male: *red box*; female: *red dots*). R2X = 0.240, Q2 = 0.071.

PCA based on these 84 targeted metabolites was used to give an intuitive display of metabolite differences. PC1 and PC2 explained 25.1% and 7.0% of the total variance, respectively (Fig. 1). The score plot showed a clear separation between AS patients and HDs but no separation between males and females. Therefore, the difference in the profiles of fecal metabolites caused by disease factor was greater than gender.

### Gender-related differences in fecal metabolites

**Figure 2:**
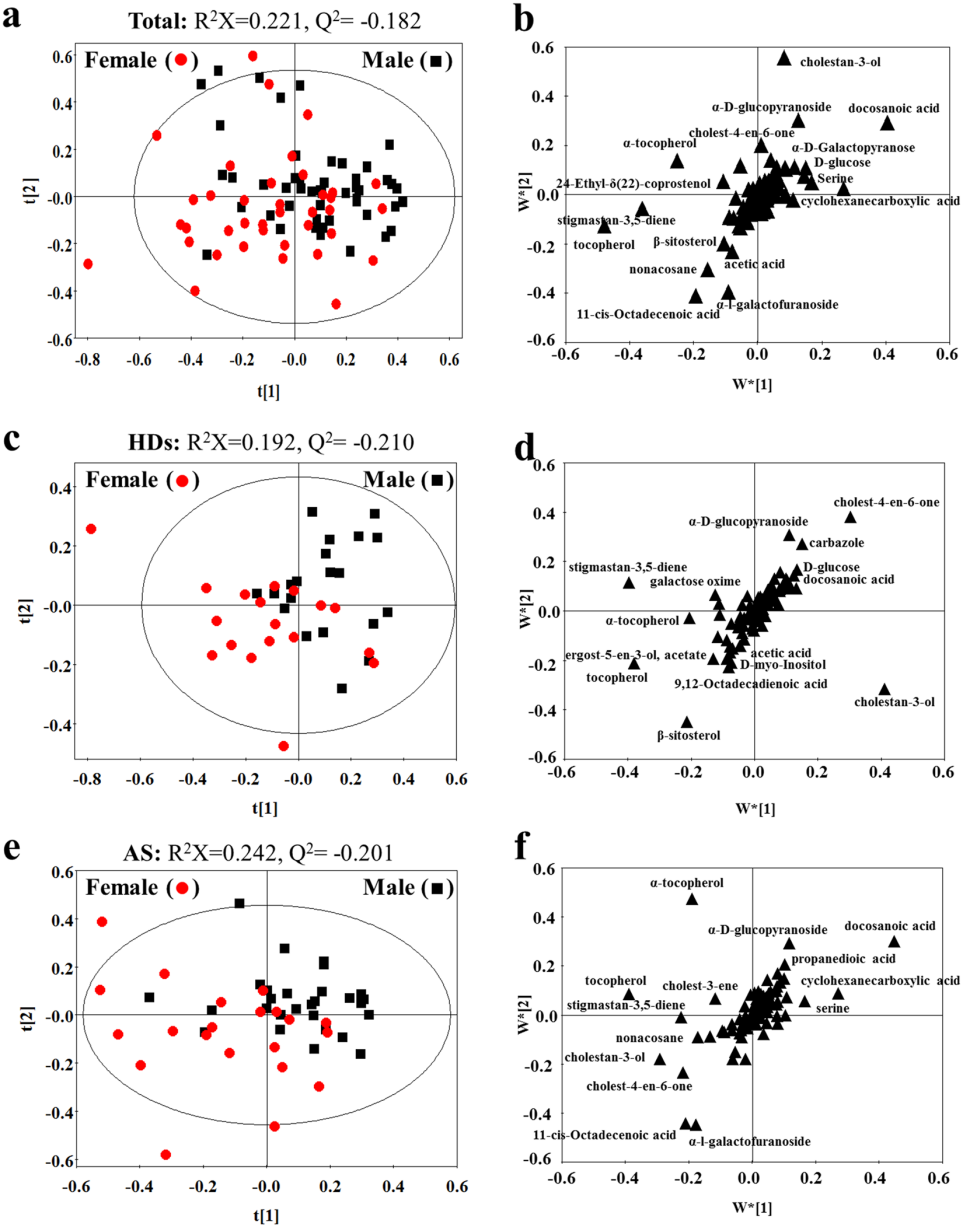
PLS-DA score (**a**,**c**,**e**) and loading plots (**b**,**d**,**f**) based on the fecal metabolic profiles of male and female individuals. (**a,b**) comparison of results between males (*black box*) and females (*red dots*) in all individuals; (**c,d**) Comparison of results between males (*black box*) and females (*red dots*) in healthy individuals (HDs); (**e,f**) comparison of results between males (*black box*) and females (*red dots*) in AS patients (AS). Metabolites with VIP > 1.0 are shown with names in loading plots. The score t1 (first component) explains the largest variation of the X space, followed by t2 (second component). The w*[1] (first component) explains the weighting variation of metabolites, followed by w*[2] (second component).

To uncover fecal metabolite differences by gender, a comparative PLS-DA model based on the targeted metabolites was conducted for males and females. The values for R^2^ and Q^2^ (Fig. 2a,c,e) and the results of permutation tests (Fig. S2) indicated poor results of the PLS-DA model in revealing differences between males and females. In addition, no clear separation between males and females was observed in PLS-DA scores (Fig. 2a,c,e). The above results indicated that there was little difference in fecal metabolites between males and females. Figures 2b,d,f shows the names of fecal metabolites with VIP > 1 in the PLS-DA.

Table 2 lists the fecal metabolites with VIP > 1 or *p* < 0.05 between genders in total subject, AS patients or HDs. The fecal metabolites with both VIP > 1 and *p* < 0.05 were considered as significantly different metabolites between males and females. There was no different metabolite between males and females in total enrolled subjects. In AS patients, a decrease in cholest-4-en-6-one and the increase in propanedioic acid were found in males compared to that of females. In healthy individuals, cholest-4-en-6-one was also the different metabolite between males and females, but its concentration was higher in males than in females. In addition, the metabolite carbazole significantly increased in males compared with females in healthy individuals. The above little differences in metabolites between males and females may be due to the inherent error in the sampling process.

**Table 2:** Significantly altered metabolites related to gender in feces extracts.

	Total — Male *vs* female			AS — Male *vs* female			HD — Male *vs* female		
	VIP	P value	Trend	VIP	P value	Trend	VIP	P value	Trend
α-Tocopherol	2.46	>0.05	—	3.47	>0.05	—	1.59	>0.05	—
Docosanoic acid	3.07	>0.05	—	3.38	>0.05	—	1.02	>0.05	—
Tocopherol	3.74	>0.05	—	3.37	>0.05	—	2.83	>0.05	—
11-cis-Octadecenoic acid	2.14	>0.05	—	2.36	>0.05	—	<1.00	>0.05	—
α-l-Galactofuranoside	1.94	>0.05	—	2.31	>0.05	—	<1.00	>0.05	—
Cholestan-3-ol	2.80	>0.05	—	2.18	>0.05	—	4.27	>0.05	—
Cyclohexanecarboxylic acid	2.15	>0.05	—	2.07	>0.05	—	<1.00	>0.05	—
Stigmastan-3,5-diene	2.87	>0.05	—	1.81	>0.05	—	3.47	>0.05	—
Cholest-4-en-6-one	1.05	>0.05	—	1.75	0.041	↓	2.55	0.028	**↑**
α-D-Glucopyranoside	1.54	>0.05	—	1.51	>0.05	—	1.60	>0.05	—
Nonacosane	1.63	>0.05	—	1.29	>0.05	—	<1.00	>0.05	—
Serine	1.30	>0.05	—	1.27	>0.05	—	<1.00	>0.05	—
Cholest-3-ene	<1.00	>0.05	—	1.14	>0.05	—	<1.00	>0.05	—
Propanedioic acid	<1.00	>0.05	—	1.12	0.020	↑	<1.00	>0.05	—
5-Hydroxyhexanoic acid diTMS	<1.00	>0.05	—	<1.0	0.039	—	<1.00	>0.05	—
D-Myo-Inositol	<1.00	>0.05	—	<1.0	0.045	—	1.08	>0.05	—
Pentacosane	<1.00	>0.05	—	<1.0	0.038	—	<1.00	>0.05	—
β-Sitosterol	1.06	>0.05	—	<<1.00	>0.05	—	2.43	>0.05	—
Carbazole	<1.00	>0.05	—	<1.00	>0.05	—	1.54	0.005	↑
Galactose oxime	<1.00	>0.05	—	<1.00	>0.05	—	1.19	>0.05	—
9,12-Octadecadienoic acid	<1.00	>0.05	—	<1.00	>0.05	—	1.19	>0.05	—
Ergost-5-en-3-ol, acetate	<1.00	>0.05	—	<1.00	>0.05	—	1.18	>0.05	—
d-Glucose	1.14	>0.05	—	<1.00	>0.05	—	1.12	>0.05	—
Acetic acid	1.14	>0.05	—	<1.00	>0.05	—	1.03	>0.05	—
9-Octadecenoic acid	<1.00	>0.05	—	<1.00	>0.05	—	<1.0	0.037	—

Note:“↑” represents significant increase in males compared with females; “↓” represents significant decrease in males compared with females; “—” represents no significant alteration between males and females.

### AS-related differences in fecal metabolites

**Figure 3:**
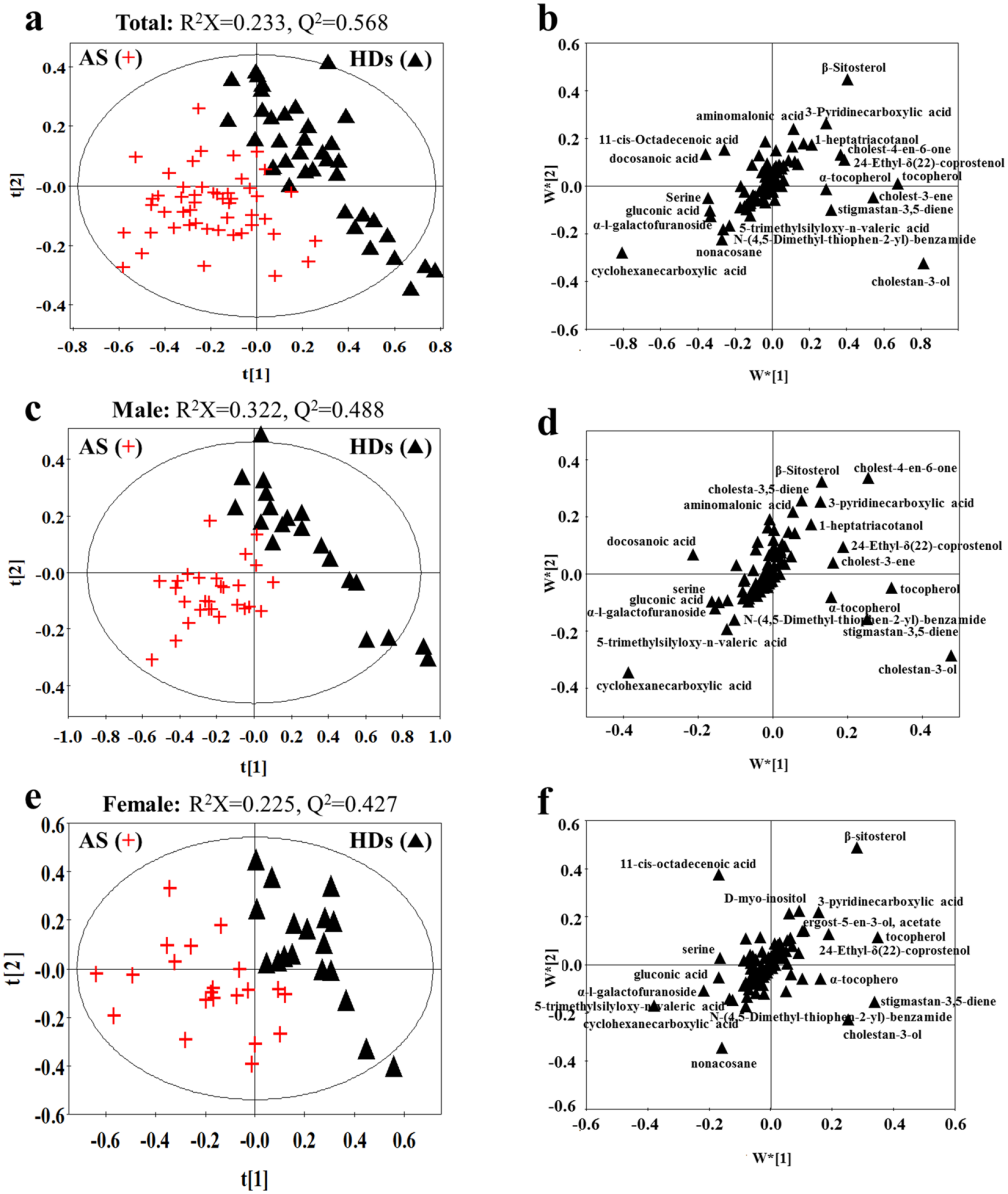
PLS-DA score (**a**,**c**,**e**) and loading plots (**b**,**d**,**f**) based on the fecal metabolic profiles of healthy individuals and AS patients. (**a**,**b**) Comparison of results between healthy (*black triangle*) and AS (*red cross*) in all individuals; (**c**,**d**) comparison of results between healthy (*black triangle*) and AS (*red cross*) in male individuals; (**e**,**f**) comparison of results between healthy (*black triangle*) and AS (*red cross*) in female individuals. Metabolites with VIP > 1.0 are shown with names in loading plots. The score t1 (first component) explains the largest variation of the X space, followed by t2 (second component). The w*[1] (first component) explains the weighting variation of metabolites, followed by w*[2] (second component).

AS-related differences in fecal metabolites were obtained by comparing AS patients and healthy individuals. First, PLS-DA analyses were used to reveal fecal signatures in three types of samples (total samples, male samples and female samples). The values for R^2^X and Q^2^ (Fig. 3a,c,e) and the results of permutation tests (Fig. S3) indicated the goodness of fit and predictability of the models in revealing different fecal metabolites between AS patients and HDs. There were clear separations between AS patients and HDs in all three PLS-DA score plots based on total, male, and female samples (Fig. 3a,c,e, respectively). Metabolites with a VIP value greater than 1.0 were displayed with name in three corresponding loading plots (Fig. 3b,d,f) and were considered the primary contributors for classification of the groups.

In combination with *p* (t-test) and VIP values, there were 19 differential metabolites between AS patients and HDs (Table 3). Nine different metabolites between AS patients and HDs were found in all three sample set. Compared with HDs, AS patients had significantly higher concentrations of 5-trimethylsilyloxy-n-valeric acid, cyclohexanecarboxylic acid, gluconic acid, serine, α-l-galactofuranoside and n-(4,5-dimethyl-thiophen-2-yl)-benzamide as well as lower concentrations of β-sitosterol, 24-ethyl-δ (22)-cloprostenol and 3-pyridinecarboxylic acid. In male subjects, the specifically different metabolites between AS patients and HDs were cholestan-3-ol, tocopherol, stigmastan-3,5-diene, cholest-3-ene, cholest-4-en-6-one and 1-heptatriacotanol, which were all significantly lower in AS patients. In female subjects, ergost-5-en-3-ol, acetate and D-myo-inositol were two distinctly different metabolites between AS patients and HDs.

**Table 3:** Significantly altered metabolites related to ankylosing spondylitis in feces extracts.

	Total—AS *vs* HD			Male—AS *vs* HD			Female—AS *vs* HD		
	VIP	P value	Trend	VIP	P value	Trend	VIP	P value	Trend
5-Trimethylsilyloxy-n-valeric acid	1.24	0.000	↑	1.22	0.000	↑	1.16	0.000	↑
Cyclohexanecarboxylic acid	3.27	0.000	↑	3.22	0.000	↑	3.10	0.004	↑
Cholestan-3-ol	3.92	0.001	↓	4.34	0.001	↓	2.51	>0.05	—
Tocopherol	2.73	0.001	↓	2.59	0.002	↓	2.87	>0.05	—
Gluconic acid	1.34	0.000	↑	1.26	0.000	↑	1.39	0.004	↑
β-Sitosterol	2.43	0.000	↓	1.73	0.006	↓	2.83	0.001	↓
Serine	1.37	0.001	↑	1.30	0.010	↑	1.42	0.026	↑
Stigmastan-3,5-diene	2.27	0.012	↓	2.31	0.001	↓	3.04	>0.05	—
24-Ethyl-δ(22)-coprostenol	1.55	0.000	↓	1.49	0.000	↓	1.56	0.009	↓
α-l-Galactofuranoside	1.35	0.000	↑	1.16	0.000	↑	1.79	0.018	↑
N-(4,5-Dimethyl-thiophen-2-yl)-benzamide	1.10	0.000	↑	1.02	0.000	↑	1.10	0.000	↑
3-Pyridinecarboxylic acid	1.54	0.000	↓	1.46	0.000	↓	1.45	0.000	↓
Cholest-3-ene	1.19	0.007	↓	1.26	0.012	↓	<1.00	>0.05	—
Docosanoic acid	1.71	0.043	↑	1.81	>0.05	—	<1.00	>0.05	—
Cholest-4-en-6-one	1.49	0.000	↓	2.36	0.000	↓	<1.00	>0.05	—
1-Heptatriacotanol	1.07	0.000	↓	1.06	0.000	↓	<1.00	0.001	—
Nonacosane	1.37	0.003	↑	<1.00	>0.05	—	1.78	>0.05	—
Ergost-5-en-3-ol, acetate	<1.00	0.007	—	<1.00	>0.05	—	1.01	0.002	↓
D-Myo-Inositol	<1.00	0.010	—	<1.00	>0.05	—	1.15	0.018	↓

Note:“↑” represents significant increase in AS patients compared with healthy individuals; “↓” represents significant decrease in AS patients compared with healthy individuals; “—” represents no significant alteration between AS patients and healthy individuals.

According to the above results, the unique fecal signatures of AS in males were cholestan-3-ol, tocopherol, stigmastan-3,5-diene, cholest-3-ene, cholest-4-en-6-one and 1-heptatriacotanol, and the unique fecal signatures of AS in females were ergost-5-en-3-ol, acetate and D-myo-inositol.

## Discussion

Intestinal disorders occurring in patients affected by ankylosing spondylitis are correlated with the severity of spine inflammation^18^. Increasing evidence has indicated that dysbiosis of the gut microbiome participates in the pathogenesis and development of AS^19,20^. Intestinal metabolites could act as crosstalk mediators in the process of gut microbiota affecting the host. There are few studies reporting that alterations of intestinal metabolites might be pathogenic factors of AS^21,22^. We have previously shown that fecal metabolites could distinguish AS patients from healthy individuals^15^. However, our previous study was limited due to the weak ability of the ^1^H-NMR platform to detect lipids and alkanes metabolites in fecal metabolomics^23^. Therefore, this study used a GC-MS metabolomics platform to explore fecal metabolites.

This study revealed the effects of gender and AS on the profiles of fecal metabolites. PLS-DA indicated that AS disease had a greater impact on fecal metabolites than gender. It has been reported that both disease and gender could affect the human fecal metabolome^24,25^. To the best of our ability, this study compared the effects of disease and gender on the fecal metabolome in the same population for the first time.

**Figure 4:**
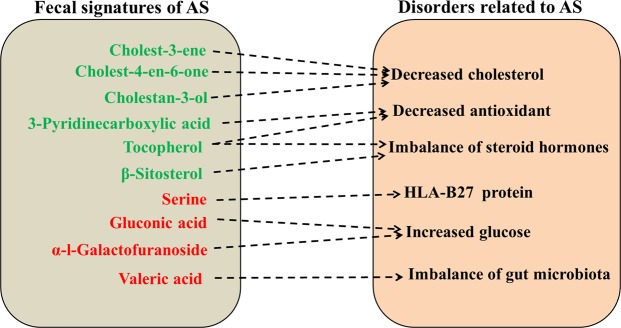
A schematic diagram showing most of fecal signatures of AS that are related to disorders of AS. *Red text* denotes increased fecal metabolites in AS patients compared with healthy individuals; *green text* denotes decreased fecal metabolites in AS patients compared with healthy individuals.

AS-related differences in fecal metabolites were the fecal signatures of AS. In this study, most of the fecal signatures of AS revealed by the GC-MS platform were lipids and alkanes, most of which were associated with the disorders of AS patients (Fig. 4). Decreased cholesterol was found in AS patients^26^, which might be associated with cholest-3-ene, cholest-4-en-6-one and cholestan-3-ol. The imbalance of steroid hormones in AS patients^27^ might be associated with decreased tocopherol^28^ and β-Sitosterol^29^. In addition, tocopherol and 3-pyridinecarboxylic acid, might cause decreased antioxidant in AS patients^30,31^. The GC-MS platform also revealed some acids as fecal signatures of AS in this study. Gluconic acid and α-l-galactofuranoside increased in the intestine, which might be associated with increased glucose in AS patients^32^. As the metabolites were derived only from gut microbiota, increased valeric acid might be due to an imbalance of gut microbiota in AS patients^33^. Serine, as the only amino acid in fecal signatures of AS revealed by GC-MS platform, was associated with misfolding of HLA-B27 protein^34^. In summary, the study enriched the types of fecal signatures of AS.

Gender had little effect on fecal metabolome, but fecal signatures of AS showed gender-specific differences. The number of fecal signatures of AS in males was greater than that in females, indicating that the fecal metabolome of males was more affected by AS disease than that in females. The different fecal signatures of AS between males and females could cause disorders of AS, such as low cholesterol, decreased antioxidants and steroid hormone imbalance. The above results might be the reason that the incidence of AS in males was higher than that in females. At the onset of AS, there were few differences in fecal metabolites between males and females, including cholest-4-en-6-one and propanedioic acid. The above different fecal metabolites might be associated with different clinical features between males and females or might be caused by inherent error in the sampling process. Certainly, the above inference requires further study to verify.

This study successfully revealed differences in the fecal signatures of AS in males and females. In addition, the study also confirmed for the first time that the effect of AS disease on fecal metabolites was greater than that of gender. However, this study failed to analyze the host’s metabolome and community structure of the gut microbiome and reveal whether the different fecal signatures of AS were derived from gut microbiome. Nevertheless, this study further indicates that AS differs between males and females and provides new directions for exploring the mechanisms of the differences in AS features between males and females.

## Supplementary information

Supplementary Materials
