# Supplementary material for: GC-MS-based fecal metabolomics reveals gender-attributed fecal signatures in ankylosing spondylitis
Source: Sci Rep. 2019 Mar 7;9:3872. doi: 10.1038/s41598-019-40351-w (PMC6405849; doi:10.1038/s41598-019-40351-w)
Supplement: Supplementary file 1 — Supplementary Materials [file 41598_2019_40351_MOESM1_ESM.docx]

**GC-MS-based fecal metabolomics reveals gender-attributed** **fecal signatures in** **ankylosing spondylitis**

Zhixing He^+^, Mingzhu Wang^+^, Haichang Li & Chengping Wen*

Institute of Basic Research in Clinical Medicine, College of Basic Medical Science, Zhejiang Chinese Medical University, Hangzhou 310053, China

Corresponding author: Address: Institute of Basic Research in Clinical Medicine, College of Basic Medical Science, Zhejiang Chinese Medical University, Hangzhou 310053, China.

Tel.: +0086-571-86613587.

*E-mail* address: wengcp@yeah.net (Chengping Wen)

**Summary of supporting information:**

**A. SUPPORTING FIGURES S1-S3**

**Figure S1** The representative total ion current (TIC) chromatogram of fecal extracts from enrolled subjects

**Figure S2** Cross-validation plots based on comparisons between male and female in AS patients (a) and healthy individuals (b).

**Figure S3** Cross-validation plots based on comparisons between AS patients and healthy individuals in total subjects (a), male subjects (b) and female subjects (c).

1. **SUPPORTING TABLES S1**

**Table S1**. List of the metabolites found in the contents of extracts of feces.


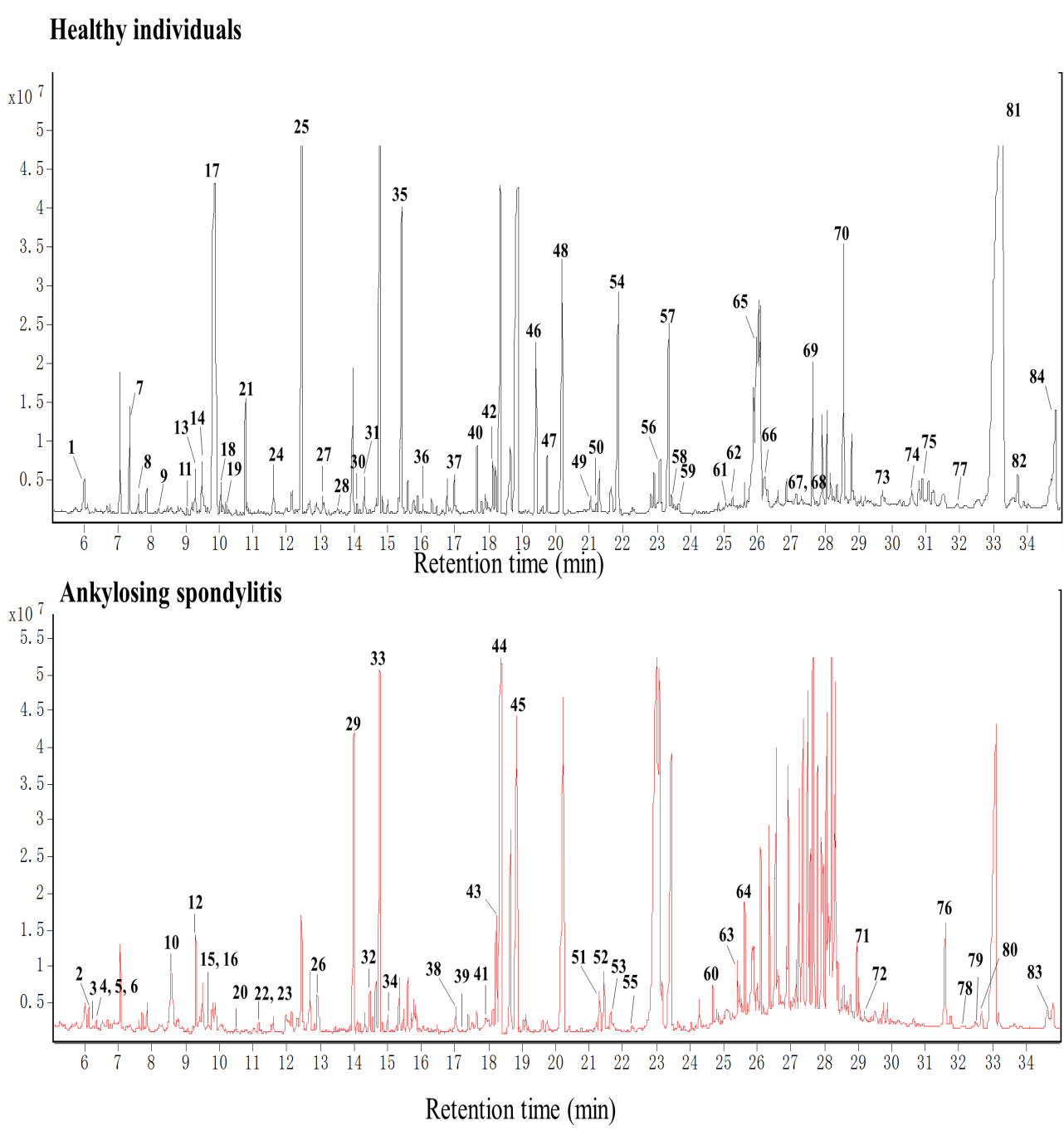


**Figure S1** The representative total ion current (TIC) chromatogram of fecal extracts from enrolled subjects


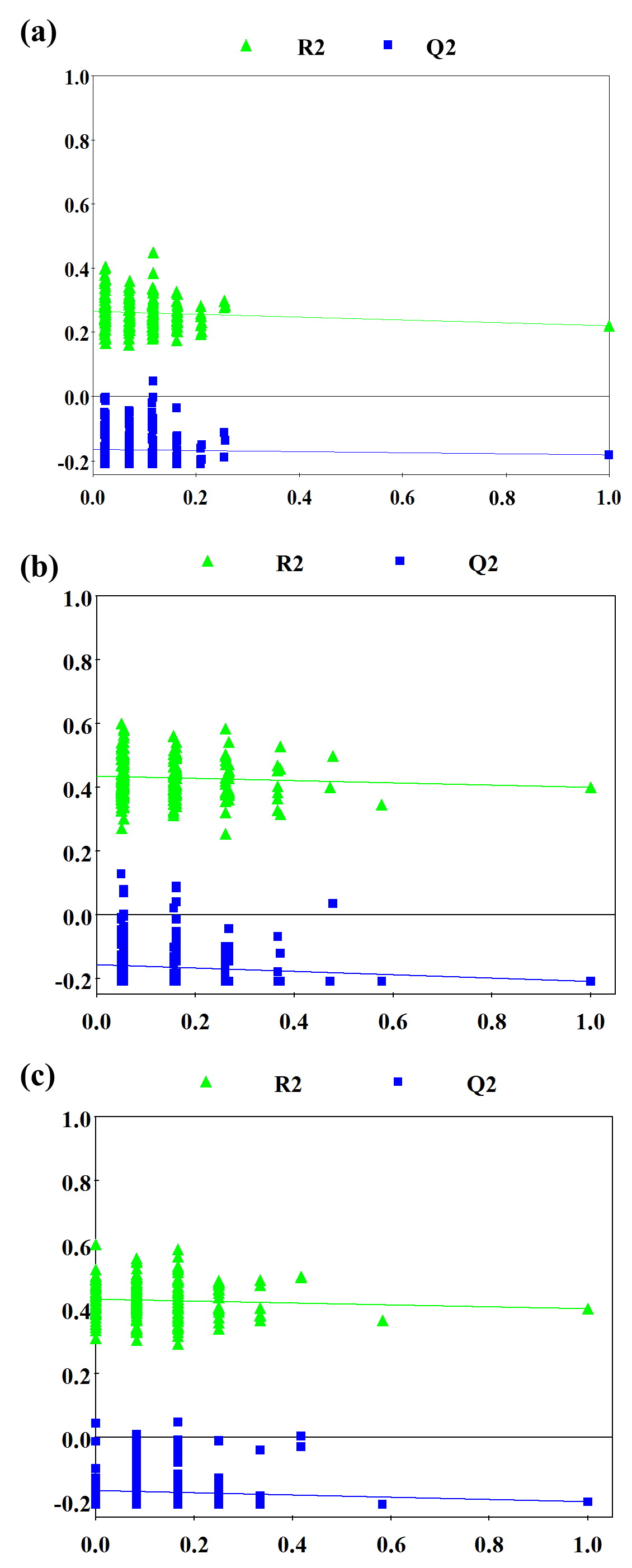


**Figure S2** Cross-validation plots based on comparisons between male and female in total subjects (a), AS patients (b) and healthy individuals (c).


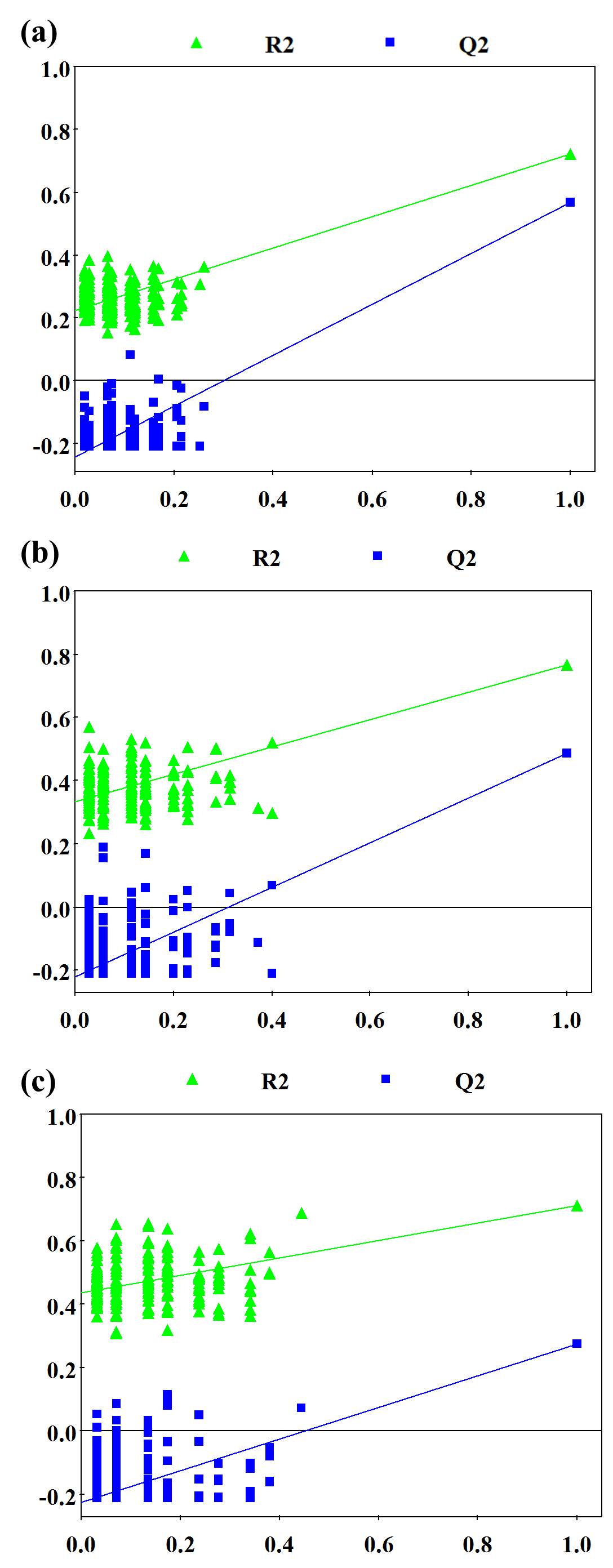


**Figure S3** Cross-validation plots based on comparisons between AS patients and healthy individuals in total subjects (a), male subjects (b) and female subjects (c).

**Table S1** List of the metabolites found in the contents of extracts of feces

| ID | Name | rt | mz | match |
| --- | --- | --- | --- | --- |
| 1 | Undecane | 5.98 | 40.10 | 870 |
| 2 | Dodecane | 6.07 | 141.10 | 886 |
| 3 | Propanoic acid | 6.25 | 118.00 | 957 |
| 4 | Hexanoic acid | 6.38 | 126.00 | 913 |
| 5 | Cyclooctane | 6.39 | 112.10 | 814 |
| 6 | Acetic acid | 6.45 | 57.10 | 952 |
| 7 | Propanedioic acid | 7.43 | 87.10 | 795 |
| 8 | Butanoic acid | 7.68 | 143.10 | 932 |
| 9 | Cyclohexanecarboxylic acid | 8.24 | 99.10 | 927 |
| 10 | Pentanoic acid | 8.75 | 145.10 | 837 |
| 11 | Octanoic acid | 9.06 | 201.10 | 780 |
| 12 | Serine | 9.20 | 132.10 | 800 |
| 13 | Glycerol | 9.31 | 189.10 | 943 |
| 14 | Silanol | 9.48 | 123.00 | 925 |
| 15 | 3-Pyridinecarboxylic acid | 9.53 | 106.00 | 701 |
| 16 | N-(4,5-Dimethyl-thiophen-2-yl)-benzamide | 9.66 | 231.10 | 709 |
| 17 | Butanedioic acid | 9.82 | 145.10 | 931 |
| 18 | 5-Trimethylsilyloxy-n-valeric acid | 10.02 | 84.10 | 932 |
| 19 | 1,1,1,3,5,7,7,7-Octamethyl-3,5-bis(trimethylsiloxy)tetrasiloxane | 10.31 | 267.00 | 790 |
| 20 | 5-Hydroxyhexanoic acid diTMS | 10.46 | 217.10 | 770 |
| 21 | Pentanedioic acid | 10.99 | 116.10 | 822 |
| 22 | 3,4-Dichloro-2,5-dicyanothiophene | 11.15 | 203.10 | 877 |
| 23 | Benzenepropanoic acid | 11.18 | 71.10 | 938 |
| 24 | Aminomalonic acid | 11.95 | 218.10 | 882 |
| 25 | cis-4-Trimethylsilyloxy-cyclohexyl(trimethylsilyl)carboxylate | 12.39 | 149.00 | 943 |
| 26 | rythronic acid | 12.94 | 206.10 | 874 |
| 27 | 7-Methoxy-2-trifluoromethyl-4-hydroxyquinoline | 13.06 | 243.20 | 930 |
| 28 | 4-Trimethylsilyloxycyclohexylacetate | 13.75 | 171.10 | 755 |
| 29 | Benzoic acid | 13.91 | 152.00 | 907 |
| 30 | Dodecanoic acid | 14.16 | 104.10 | 715 |
| 31 | Gluconic acid | 14.46 | 201.10 | 838 |
| 32 | d-Ribose | 14.53 | 219.10 | 866 |
| 33 | Cyclooctene | 14.71 | 101.00 | 710 |
| 34 | Heptadecane | 14.99 | 198.20 | 846 |
| 35 | D-Galactose | 15.58 | 126.10 | 740 |
| 36 | α-l-Galactofuranoside | 16.12 | 217.10 | 743 |
| 37 | Benzeneacetic acid | 16.99 | 148.10 | 922 |
| 38 | Tetradecanoic acid | 17.03 | 156.10 | 906 |
| 39 | 1-Trimethylsilyloxypentadecane | 17.21 | 58.10 | 877 |
| 40 | Myo-inositol | 17.81 | 193.10 | 916 |
| 41 | D-Fructose | 17.90 | 223.10 | 900 |
| 42 | n-Pentadecanoic acid | 18.17 | 96.10 | 838 |
| 43 | Eicosane | 18.22 | 197.20 | 835 |
| 44 | d-Glucose | 18.37 | 114.00 | 901 |
| 45 | Hexadecane | 18.88 | 169.20 | 851 |
| 46 | Inositol | 19.24 | 149.10 | 823 |
| 47 | α-D-Galactopyranose | 19.60 | 191.10 | 932 |
| 48 | Hexadecanoic acid | 20.22 | 297.20 | 912 |
| 49 | 9-Octadecenoic acid | 21.02 | 98.10 | 740 |
| 50 | Heptadecanoic acid | 21.14 | 117.00 | 908 |
| 51 | N-Acetyl glucosamine methoxime | 21.31 | 303.20 | 819 |
| 52 | Octadec-9Z-enol trimethylsilyl ether | 21.51 | 340.30 | 733 |
| 53 | Pentacosane | 21.62 | 155.20 | 840 |
| 54 | Linoleic acid | 22.02 | 262.20 | 793 |
| 55 | Galactose oxime | 22.37 | 139.10 | 780 |
| 56 | 11-cis-Octadecenoic acid | 23.15 | 222.20 | 932 |
| 57 | Octadecanoic acid | 23.47 | 329.25 | 930 |
| 58 | Octanedioic acid | 23.55 | 344.25 | 726 |
| 59 | 9,12-Octadecadienoic acid | 23.62 | 191.10 | 898 |
| 60 | Nonacosane | 24.84 | 57.10 | 844 |
| 61 | Arachidonic acid | 25.07 | 106.10 | 814 |
| 62 | Tetradecane | 25.25 | 85.10 | 731 |
| 63 | Carbazole | 25.45 | 365.30 | 885 |
| 64 | D-Myo-Inositol | 25.88 | 305.20 | 840 |
| 65 | β-Sitosterol | 25.98 | 158.10 | 827 |
| 66 | Pentadecanoic acid | 26.15 | 225.20 | 731 |
| 67 | Docosanoic acid | 27.14 | 117.00 | 732 |
| 68 | Cholest-4-en-3-one | 27.58 | 385.30 | 830 |
| 69 | α-D-Glucopyranoside | 27.63 | 437.20 | 890 |
| 70 | D-Turanose | 28.54 | 318.20 | 808 |
| 71 | D-Mannitol | 29.01 | 308.20 | 726 |
| 72 | Maltose | 29.25 | 243.10 | 730 |
| 73 | Cholesta-3,5-diene | 29.71 | 79.00 | 921 |
| 74 | Tocopherol-ç-tms-derivative | 30.66 | 488.50 | 862 |
| 75 | Ergost-5-en-3-ol, acetate | 30.94 | 382.40 | 725 |
| 76 | Cholest-3-ene | 31.70 | 132.10 | 734 |
| 77 | Cholestan-3-ol | 31.95 | 75.00 | 900 |
| 78 | Stigmastan-3,5-diene | 32.17 | 43.10 | 782 |
| 79 | Cholesterol | 32.48 | 228.20 | 949 |
| 80 | α-Tocopherol (vitamin E) | 32.72 | 502.50 | 867 |
| 81 | Cholest-4-en-6-one | 33.28 | 231.20 | 720 |
| 82 | 24-Ethyl-δ(22)-coprostenol | 33.70 | 374.30 | 751 |
| 83 | 3-α-Trimethylsilyloxycholanic acid | 34.75 | 463.30 | 816 |
| 84 | 1-Heptatriacotanol | 34.89 | 262.20 | 703 |
